# Oxidative, Reductive, and Nitrosative Stress Effects on Epigenetics and on Posttranslational Modification of Enzymes in Cardiometabolic Diseases

**DOI:** 10.1155/2020/8819719

**Published:** 2020-10-31

**Authors:** I. Pérez-Torres, M. E. Soto, V. Castrejón-Tellez, M. E. Rubio-Ruiz, L. Manzano Pech, V. Guarner-Lans

**Affiliations:** ^1^Cardiovascular Biomedicine Department, Instituto Nacional de Cardiología “Ignacio Chávez”, Juan Badiano 1, Sección XVI, Tlalpan, Mexico City 14080, Mexico; ^2^Immunology Department, Instituto Nacional de Cardiología “Ignacio Chávez”, Juan Badiano 1, Sección XVI, Tlalpan, Mexico City 14080, Mexico; ^3^Physiology Department, Instituto Nacional de Cardiología “Ignacio Chávez”, Juan Badiano 1, Sección XVI, Tlalpan, Mexico City 14080, Mexico

## Abstract

Oxidative (OS), reductive (RS), and nitrosative (NSS) stresses produce carbonylation, glycation, glutathionylation, sulfhydration, nitration, and nitrosylation reactions. OS, RS, and NSS are interrelated since RS results from an overactivation of antioxidant systems and NSS is the result of the overactivation of the oxidation of nitric oxide (NO). Here, we discuss the general characteristics of the three types of stress and the way by which the reactions they induce (a) damage the DNA structure causing strand breaks or inducing the formation of 8-oxo-d guanosine; (b) modify histones; (c) modify the activities of the enzymes that determine the establishment of epigenetic cues such as DNA methyl transferases, histone methyl transferases, acetyltransferases, and deacetylases; (d) alter DNA reparation enzymes by posttranslational mechanisms; and (e) regulate the activities of intracellular enzymes participating in metabolic reactions and in signaling pathways through posttranslational modifications. Furthermore, the three types of stress may establish new epigenetic marks through these reactions. The development of cardiometabolic disorders in adult life may be programed since early stages of development by epigenetic cues which may be established or modified by OS, RS, and NSS. Therefore, the three types of stress participate importantly in mediating the impact of the early life environment on later health and heritability. Here, we discuss their impact on cardiometabolic diseases. The epigenetic modifications induced by these stresses depend on union and release of chemical residues on a DNA sequence and/or on amino acid residues in proteins, and therefore, they are reversible and potentially treatable.

## 1. Introduction

Mammalian cells contain two different genomes: the nucleic genome and a smaller mitochondrial genome, and both genomes may be epigenetically modified. Many environmental factors and signals derived from metabolic pathways serve as messengers to coordinate the genetic response and to communicate both genomes [1–3]. Oxidative (OS), reductive (RS), and nitrosative (NSS) stresses are among the signals that affect the structure of DNA both in the nucleus and the mitochondria. The three types of stress also modify proteins including histones, enzymes participating in the establishment of the classical epigenetic cues, DNA damage reparation enzymes, and proteins participating in intracellular pathway through posttranslational regulation. They also induce changes that may act as new epigenetic marks. Therefore, these stresses participate in the nongenomic tuning of the phenotype modifying previously existing epigenetic cues, having beneficial effects, or increasing or decreasing the risk of diseases later in life including cardiometabolic disorders such as obesity, metabolic syndrome, diabetes, hypertension, atherosclerosis, and cardiac hypertrophy. The development of these diseases may be programed by epigenetic cues modified by OS, RS, and NSS from early stages of development. Therefore, the three types of stress mediate the impact of the early life environment on later health and its heritability [4, 5].

**Figure 1:**
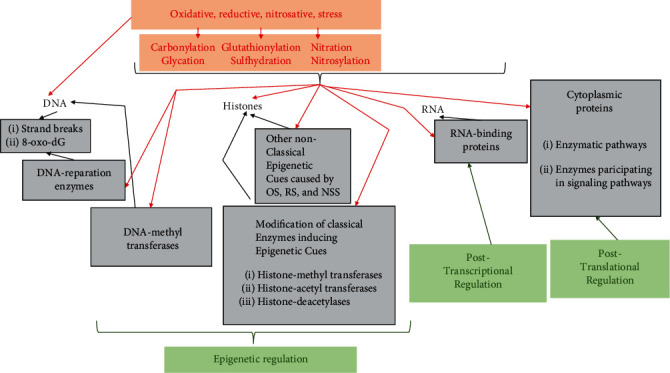
Mechanisms and levels at which oxidative, reductive, and nitrosative stress produce cell damage. Mechanisms (in red) include the direct action on DNA and on proteins. The levels at which oxidative, reductive, and nitrosative stress act (in green) include epigenetic regulation, posttranscriptional regulation, and posttranslational regulation.

The most common epigenetic cues are DNA methylation, histone modifications (methylation and acetylation), and noncoding RNAs. However, other mechanisms induced by OS, RS, and NSS may also modify the structure or reading of DNA, alter the histones upon which the DNA is structured, or modify the enzymes involved in DNA reparation. Reactions such as carbonylation, glycation, glutathionylation, sulfhydration, nitration, and nitrosylation may also act as epigenetic cues (see Figure 1).

**Figure 2:**
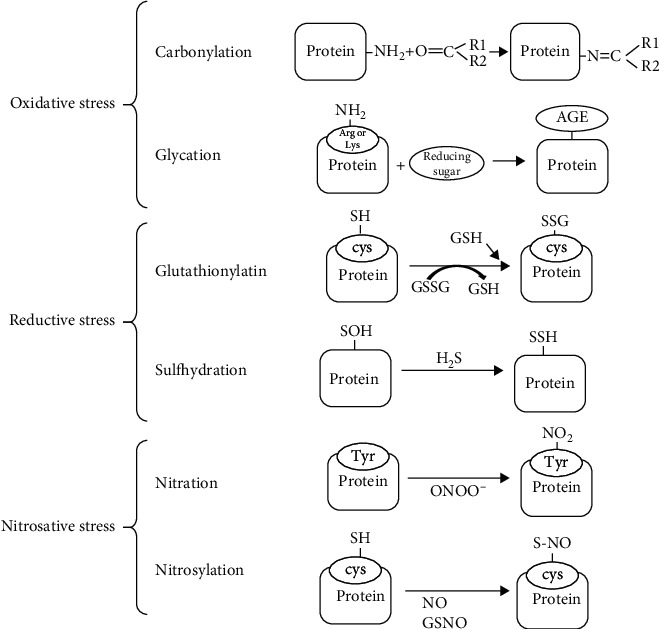
Chemical reactions induced by oxidative, reductive, and nitrosative stress in proteins. These include carbonylation, glycation, glutathionylation, sulfhydration, nitration, and nitrosylation.

Epigenetic modifications induced by OS, RS, and NSS depend on union and release of chemical residues on a DNA sequence and/or on amino acid residues in histones in the same way as classical epigenetic cues, and therefore, they are reversible and potentially treatable [6, 7]. Thus, although epigenetic inheritance resembles genetic inheritance of DNA in the capacity to transmit acquired characteristics through generations, epigenetic classic mechanisms differ in their capacity to be reversible through changes in the environment and by variations in nutritional factors. The mechanisms and the cellular levels at which OS, RS, and NSS act are illustrated in Figure 2 and will be discussed throughout this review. In this paper, we also discuss the epigenetic cues that may be induced by OS, RS, and NSS and their impact on cardiometabolic diseases.

## 2. Definition and Mediators of Oxidative, Reductive, and Nitrosative Stress

### 2.1. Oxidative Stress

Reactive oxygen species (ROS) are oxygen-containing molecules with an uneven number of electrons that allow them to react rapidly with other molecules. ROS include the radical superoxide anion (O_2_^−^), hydroxyl radicals (OH^·^), alkoxyl radicals (RO^·^), peroxyradicals (ROO^·^), hydrogen peroxide (H_2_O_2_), hypochlorous acid (HOCl), and the oxygen singlet (^1^O_2_) [8]. Free radicals can cause large-chain chemical reactions because they easily react with other molecules inducing oxidation that may be beneficial or harmful. When functioning properly, they help fight off pathogens which lead to infections and function as second messengers. However, they may also constitute a source of cellular damage [9].

ROS are produced at several intracellular locations, including mitochondria, peroxisomes, plasma membrane, endoplasmic reticulum, and cytoplasm. ROS are mainly generated by mitochondria through oxidative phosphorylation and the activity of the mitochondrial complexes I and III [10]. Mitochondrial ROS are the principal species that cause peroxidation of polyunsaturated fatty acids localized at cellular membranes as well as DNA. In DNA, they promote single- and double-strand breaks, and in protein, they cause damage through oxidation of sulfhydryl and aldehyde groups, protein-protein interactions, and fragmentation [11, 12].

Since most mitochondrial proteins are encoded in the nuclear genome, there must exist a suitable communication between nucleus, cytoplasm, and mitochondrial compartments which is essential for maintaining appropriate mitochondrial function. Human mitochondrial DNA is a 16.5 kb circular double-stranded DNA containing a heavy and a light strand and located in the mitochondrial matrix. It contains 37 genes encoding for 13 subunits of the oxidative phosphorylation complexes I, III, IV, and V; two ribosomal RNAs; and 22 transfer RNAs. The replication, transcription, and maintenance of mitochondrial DNA are carried out by a nuclear-encoded factor [13].

ROS are also produced by cytosolic enzymes such as the reduced nicotinamide adenine dinucleotide phosphate (NADPH) oxidase, cyclooxygenases, xanthine oxidase, and cytochrome P450. The decoupling of the endothelial nitric oxide synthase (eNOS) also produces ROS. These enzymes are implicated in the redox balance [14].

### 2.2. Reductive Stress

RS is the counterpart of OS. It is the result of an elevated level of thiol groups that leads to an increase of reducing equivalents such as NADPH, glutathione (GSH)/glutathione disulfide (GSSG) ratio or to an elevated level of sulfhydric acid (H_2_S) [15]. GSH is constituted by glutamate, cysteine, and glycine. It is synthetized by γ-glutamyl-cysteine synthetase (GCL) and GSH synthetase, and it is regenerated by glutathione reductase (GR) [8]. GSH acts as a second messenger in cells and is the most abundant endogenous intracellular antioxidant. Although it is mostly found in a free form (85%), it can also bind to proteins [16]. GSH regenerates vitamins E and C and is able to inactivate O_2_^−^ and OH^·^ radicals [17]. GSH is oxidized to GSSG in the presence of ROS, and, in turn, GSSG is recycled back to GSH, by GR. This reaction is NADPH-dependent [18].

GSH is a source of reducing equivalents that are important for the proper function of the glutathione peroxidase (GPx) isoforms [19]. GPx are a family of homologous enzymes that contain a selenium-cysteine that is an important antioxidant enzyme involved in preventing the harmful accumulation of intracellular H_2_O_2_. An increase in the GSH levels leads to abnormal mitochondrial oxidation and disruption of mitochondrial homeostasis [20].

Sulfhydric acid (H_2_S) is also a very potent reducing agent. H_2_S donors modify thiol groups of specific cysteines in target proteins via sulfhydration. This gas is a pleiotropic transmitter, which is produced from L-cysteine in the intestine by the sulfate-reducing bacteria as an end product of anaerobic respiration [21, 22].

H_2_S is also endogenously produced by multiple transsulfuration reactions catalyzed by the enzymes cystathionine gamma-lyase (CSE), cystathionine *β*-synthase (CBS), and 3-mercaptopyruvate sulfurtransferase (3MST) [23]. These enzymes are not only present in the gut but are also found in other organs, having a tissue-specific rate of expression.

H_2_S is a biologically relevant signaling molecule with potential roles in several physiological processes and plays versatile roles in cell death/survival [24]. H_2_S can penetrate plasma membranes and transduce intracellular and intercellular signals without the need of a receptor [21].

**Figure 3:**
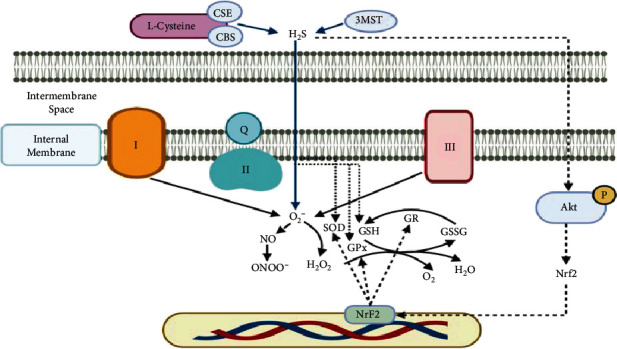
H_2_S as a means for generating H_2_O_2_ thus altering cell signaling processes and activating transcription factors such as the nuclear factor erythroid 2-related factor 2 (Nrf2), which regulates the expression of antioxidant proteins. Abbreviations: CSE: cystathionine gamma-lyase; CBS: cystathionine *β*-synthase; 3MST: 3-mercaptopyruvate sulfurtransferase; H_2_S: sulfhydric acid; O_2_^−^: superoxide; SOD: superoxide dismutase; H_2_O_2_: hydrogen peroxide; GPx: glutathione peroxidase; GR: glutathione reductase; GSH: glutathione; GSSG: oxidized glutathione; NO: nitric oxide; ONOO^−^: peroxynitrite.

Polysulfides act as catalysts for sulfide oxidation and the concomitant production of ROS. However, the H_2_O_2_ produced in the autoxidation of H_2_S may be relevant for cell signaling. Thus, H_2_S serves as a means for generating H_2_O_2_ in cell signaling processes under some circumstances, and it activates transcription factors such as the nuclear factor erythroid 2-related factor 2 (Nrf2), which regulates the expression of antioxidant proteins [25] (see Figure 3).

### 2.3. Nitrosative Stress

Reactive nitrogen species (RNS) are the result of an increased oxidation of nitric oxide (NO). NO is produced by the nitric oxide synthase (NOS) isoforms and has important physiological actions constituting an important vasodilator and neurotransmitter. Elevated levels of RNS are caused by an excess production of NO by the inducible nitric oxide synthase (iNOS) or by the uncoupled eNOS in the presence of high concentrations of O_2_^−^ [8]. At low concentrations, NO protects the cells from proapoptotic effects, but elevated levels lead to apoptosis [26]. RNS, in proper concentrations, function as second messengers, participate in signal transduction pathways, and serve as nonspecific defenses forming part of the immune responses. However, when their concentrations are increased, RNS elevate the level of toxic molecules and may induce cellular damage in the presence of an oxidative environment [27, 28]. The following molecules are considered RNS: peroxynitrite (ONOO^−^), nitrogen dioxide (^·^NO_2_), peroxynitrous acid (HNO_3_), dinitrogen trioxide (N_2_O_3_), nitroxyl (HNO), peroxynitrous acid (ONOOH), peroxynitrate (O_2_NOO^−^), peroxynitric acid (O_2_NOOH), nitrosonium cation (NO^+^), nitrate (NO_3_^−^), nitrite (NO_2_^−^), and nitroxyl anion (NO^−^) [8].

### 2.4. Protection against Stress

Oxidative stress results from an imbalance between free radicals and antioxidants in the body. Antioxidants are molecules that can donate an electron to a free radical without making themselves unstable. This causes the free radical to stabilize and become less reactive. Oxidation is a normal and necessary process that takes place in cells; however, when there are more free radicals present than can be kept in balance by antioxidants, the free radicals start damaging fatty cell components, DNA, and proteins [29].

Enzymatic and nonenzymatic antioxidant systems regulate the level of ROS to remain at a functional level. The enzymatic system includes superoxide dismutase (SOD) with its three isoforms: copper-zinc (Cu-ZnSOD), manganese SOD (MnSOD), and extracellular (ECSOD); catalase (CAT), enzymes that employ glutathione (GSH) such as GPx isoforms, glutathione-S-transferase (GST), GR, glutaredoxin (Grd), and peroxythioredoxins (Trx) [30]. The nonenzymatic antioxidant systems are represented by reduced GSH, ascorbic acid (vitamin C), *α*- and *β*-tocopherol (vitamin E), *α*-retinol, (vitamin A), lycopene, ubiquinol-10, carotene, water soluble uric acid, pyruvate, and bilirubin [31]. An excess of antioxidants can also lead to a redox imbalance [32].

## 3. Oxidative Stress and Changes in Nuclear and Mitochondrial DNA

Oxidative stress may result in genome instability and global heterochromatin loss thus affecting the chromatin states [33–35]. Chromatin can be found in the next four states: (1) transcriptionally active chromatin; (2) repressed chromatin; (3) silent chromatin, which is not associated with specific proteins or histone marks; and (4) heterochromatin protein 1- (HP1-) associated chromatin [36]. These different states are important to maintain proper transcription [37, 38], mitosis [39], and meiosis [40].

Different mechanisms protect cells from heterochromatin loss and enhance genome stability upon exposure to OS. When exposed to H_2_O_2_, there is an increase in the stability of pericentromeric heterochromatin which has elevated levels of the histone H3K9 methyltransferase SUV39H1, SIRT1 deacetylase, and HP1 proteins [41].

Damage in mitochondrial DNA may lead to a decrease in the expression of electron transport chain components or in the expression of components that produce more ROS. These damages in mitochondrial DNA also correlate with diseases including atherosclerosis. This has been found in mouse models and human tissues. Damage to mitochondrial DNA plays an important role in different diseases including diabetes, obesity, dyslipidemia, hypertension, arrhythmias, and sudden cardiac death. Investigations developed by different groups have exposed a complex association between environmental factors, mitochondrial metabolism, epigenetic signals, and transcriptional programs. Mitochondria do not have specific DNA reparation enzymes, and this genome is therefore more vulnerable to damage by these three types of stress [42].

### 3.1. Effects of DNA Damage on Gene Expression and on DNA Reparation Processes

DNA can be directly affected by OS which causes strand breaks. Deoxyguanosine (dG) is the most prone nucleoside toward oxidation, and it is turned into 8-oxo-d guanosine (8-oxo-dG) by ROS. This end product is regularly bound and excised by 8-oxoguanine DNA glycosylase and then repaired by the base excision repair-pathway. When the oxidative damage is important, reparation mechanisms may not be able to compensate for the damage [43].

Damaged bases, as a consequence of OS, can contribute to gene regulation. The preferred targets for oxidation are G-quadruplex (G4) that are guanine tetrads which are stabilized by hydrogen bonds and monovalent cations. Some transcription factors are usually bound to these sequences and the alterations of the G4 conformational state participate in the regulation of gene expression [44]. Inactive 8-Oxoguanine DNA glycosylase binds to 8-oxo-dG, recruiting transcription factors and enhancing gene expression [45]. Oxidative DNA damage can also inhibit binding of other chromatin proteins [46].

GSH, one of the main molecules that induce RS, is recognized as an agent that induces DNA damage and impairs repair mechanism, redox regulation, and cell signaling pathways [47, 48].

H_2_S is a DNA-damaging mutagen generating single-strand DNA cleavage. This process involves autoxidation of H_2_S to generate O_2_^−^, H_2_O_2_, and ^·^OH that damage DNA via a trace metal-mediated Fenton-type reaction [49]. Even if cells contain little or no free transition metals, protein-bound metals can participate in redox processes [50]. At a physiological pH, there are significant amounts of sulfur anion HS^−^, which is the principal substrate for aerobic oxidation [51].

The increase in the cleavage of the DNA strand with increasing H_2_S concentrations may favor the formation of elemental sulfur that may react with HS^−^ to generate polysulfides that, in turn, react with O_2_ to generate additional O^2-^ [52].

**Figure 4:**
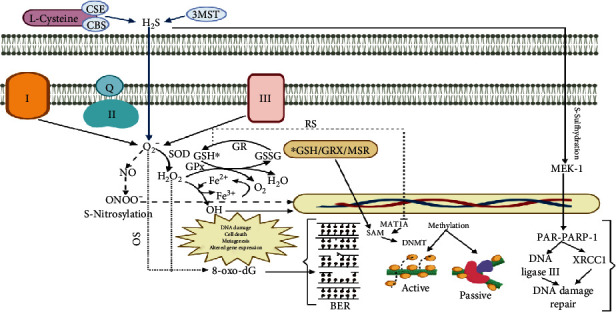
Damage induced to DNA by oxidative, reductive, and nitrosative stress. Abbreviations: CSE: cystathionine gamma-lyase; CBS: cystathionine *β*-synthase; 3MST: 3-mercaptopyruvate sulfurtransferase; H_2_S: sulfhydric acid; O_2_^−^: superoxide; SOD: superoxide dismutase; H_2_O_2_: hydrogen peroxide; OH^·^: hydroxyl radical; GPx: glutathione peroxidase; GR: glutathione reductase; GSH: glutathione; GRX: glutaredoxin; MSR: methionine sulfoxide reductase; GSSG: oxidized glutathione; NO: nitric oxide; ONOO: peroxynitrite; PARP-1: polymerase 1; OS: oxidative stress; RS: reductive stress; 8-oxo-dG: 8-oxo-d guanosine; BER: base excision repair; DNMT: DNA methyltransferases; SAM: S-adenosylmethionine; MAT1A: methionine adenosyltransferase 1A.

Sulfhydration leads to poly [ADP-ribose] polymerase 1 (PARP-1) activation through direct interaction. PARP-1 detects DNA damage and helps select the repair pathway needed. Upon damage on DNA, PARPs bind to DNA strand breaks and catalyze the addition of long branched chains of PARs onto themselves and other chromatin remodeling factors. PARPs transfer ADP-ribose from NAD^+^ to glutamic acid residues on a protein acceptor and/or on themselves, allowing the formation of ADP-ribose polymers (PARs). In the presence of H_2_S, activated PARP-1 recruits XRCC1 and DNA ligase III to DNA breaks to mediate DNA damage reparation [23, 50] (see Figure 4).

Nitrosative stress interacts with nucleic acids and forms 8-oxo-dG and 8-nitroguanidine which can cause breaks in DNA and formation of single-strand DNA [53]. NO and NSS also participate in the control of the structure of chromosomes [54]. Chromatin modification can be associated with some of the chemical reactions of NO and its metabolic processes including S-nitrosylation of thiols, tyrosine nitration, and cGMP production (see Figure 4).

## 4. Oxidative, Reductive, and Nitrosative Stress and Posttranslational Modifications to Proteins including Histones

Posttranslational modifications (PTMs) by OS, RS, and NSS are essential mechanisms to diversify protein functions and coordinate signaling networks [55]. The three types of stress act on histones, enzymes participating in the establishment of epigenetic cues, DNA reparation proteins, and proteins in the cytosol acting on enzymatic and signaling pathways. They control protein folding, protein targeting to different subcellular compartments, protein interaction, and functional state, including the catalytic activity of enzymes and of signaling in transduction pathways. Some PTMs are easily reversible by the action of deconjugating enzymes. Modifying and demodifying enzymes by PTM permit a rapid and not expensive regulation of protein function that take less time and imply a smaller expense of bioenergy [56]. As an example, RNS can S-nitrosylate thiols to modify key signaling molecules such as kinases and transcription factors. Several key enzymes in mitochondrial respiration are also inhibited by nitrogen species leading to a depletion of ATP and cellular energy [57].

In addition to the participation of the three types of stress on posttranslational modifications of proteins, they may also affect posttranscriptional processes through the regulation of RNA-binding proteins (RBPs). Genetic information stored in chromosomal DNA is translated into proteins through mRNAs. When pre-mRNAs are transcribed by RNA polymerase II in the nucleus, they undergo several PTMs induced by RBPs. These processing steps regulate the fate of the transcript [58]. Posttranscriptional gene expression is regulated by RBPs which intervene in multiple cellular processes. Moreover, different classes of RBPs interact with various small noncoding RNAs to form ribonucleoprotein complexes that participate in many aspects of cell metabolism, such as DNA replication, expression of histone genes, regulation of transcription, and translational control [58].

### 4.1. Reactions by Which Oxidative Stress Modifies Posttranslational Regulation

ROS react with proteins including histones, enzymes determining the establishment of epigenetic cues, enzymes participating in intracellular signaling pathways, and enzymes responsible for the reparation of DNA damage resulting in oxidative modifications that include cleavage of the polypeptide chain, hydroxylation of aromatic groups and aliphatic amino acid side chains, formation of protein hydroperoxides, oxidation of methionine residues, oxidation of sulfhydryl groups, conversion of some amino acid residues into carbonyl groups, and formation of cross-linking bonds that give rise to large aggregates. Oxidative modification occurs particularly in aromatic and sulfur-containing residues [59–62].

Protein carbonylation is an irreversible PTM by which a reactive carbonyl group such as an aldehyde, ketone, or lactam is incorporated into the structure of a protein (Figure 2). Protein-bound carbonyls are derived from metal-catalyzed oxidation that results from the Fenton reaction and generate highly reactive OH^·^ [64]. OH^·^ can, in turn, oxidize amino acid side chains or break the protein backbone, resulting in many alterations such as reactive carbonyls [65]. Oxidation of proline and arginine leads to the generation of glutamic semialdehyde; lysine is oxidized to aminoadipic semialdehyde and threonine to 2-amino-3-ketobutyric acid [66]. Oxidation of tryptophan by ROS gives rise to more than seven oxidation products [67]. Carbonyls derived from reactive lipid peroxidation products also bind to proteins [68–70].

Glycoxidation also results in protein carbonylation where reactive *α*-carbonyls such as glyoxal, methylglyoxal, and 3-deoxyglucosone that are formed during glycoxidation modify lysine and arginine residues to generate pyrralines and imidazolones among other products [71, 72].

The reaction of reducing sugars such as glucose or fructose with the side chains of lysine and arginine residues, known as glycation, forms Amadori and/or Hynes products that can be further decomposed by ROS into advanced glycation end products (AGE) that can contribute to protein carbonylation [73].

ROS also modify histones, and since histones are the most common chromatin proteins, alterations in their abundance, structure, or PTMs will have a severe impact on the global structure of chromatin, influencing gene expression, genome stability, and replication [4]. Histone modifications induced by ROS include changes in methylation, acetylation, ubiquitylation, ADP-ribosylation, SUMOylation, and phosphorylation, leading to their altered folding, stability, and ability to be posttranslationally modified [74].

Oxidized proteins are toxic decreasing cellular viability and are therefore repaired or removed from cells [75, 76]. Proteins damaged by OS are proteolyzed in a ubiquitin- and ATP-independent way.

### 4.2. Mechanism by Which Reductive Stress Acts on Posttranslational Regulation

RS impairs cellular functions, and GSH can be viewed as a new posttranslational modifier of proteins and of histones. The ratio of the GSH/GSSG redox couple regulates S-glutathionylation in cells [47]. S-Glutathionylation is a potent mechanism for posttranslational modulation of a variety of regulatory and metabolic proteins when there is a change in the cellular redox status (lower GSH/GSSG ratio). S-Glutathionylation occurs in protein cysteine residues by the addition of glutathione [77] (Figure 2).

Furthermore, under RS, the relatively oxidizing environment that is needed in the endoplasmic reticulum for the proper formation of disulfide bonds of membrane and secretory proteins is lost. Therefore, protein disulfide bonds are not normally formed, resulting in activation of the unfolded protein response and endoplasmic reticulum stress [78].

Thiol–disulfide homeostasis loss is an important consequence of many diseases. Any modification of critical cysteine residues on enzymes, receptors, transport proteins, and transcription factors is recognized as an important mechanism of signal transduction perturbation [47].

Glutathionylation of DNA-repair proteins may lead to a failure in the reparation mechanisms. An elevated level of GSH leads to the deglutathionylation of DNA-repair proteins [4].

GSH is also considered a posttranslational modifier of histones altering the structure of the nucleosome. GSH links metabolism to the control of epigenetic mechanisms at different levels including substrate availability, enzymatic activity for DNA methylation, and alterations in the expression of microRNAs. It has been speculated that mutations in enzymes involved in GSH metabolism and the alterations of the levels of cofactors affecting epigenetic mechanisms might be connected [79].

### 4.3. Mechanisms by Which Nitrosative Stress Acts on Posttranslational Regulation of Proteins

ONOO^−^ modifies proteins via S-nitrosylation at cysteine residues modifying their function (Figure 2). Nitrated cysteines are the main alteration participating in redox signaling events. Most proteins contain cysteine residues which are the second most abundant amino acid in the proteins (1.9%). Nevertheless, ONOO^−^ can only modify a small percentage of these cysteines [80].

S-Nitrosylation is a nonenzymatic reversible reaction, in which a covalent NO is attached to a reactive cysteine residue to form S-nitrosothiols (SNOs), such as low-molecular-weight S-nitrosoglutathione (GSNO) and S-nitrosylated proteins. The S-nitrosylation may happen by transnitrosylation that involves an acceptor thiol and GSNO [81]. GSNO, is the main endogenous SNO, serving as a stable reservoir of intracellular NO. The main denitrosylating enzyme is S-nitrosoglutathione reductase (GSNOR). The activity of this enzyme is important in the regulation of SNO action [82]. GSNOR metabolizes GSNO and therefore depletes the levels of S-nitrosylated protein, which are in equilibrium with GSNO [83]. When the activity of GSNOR is decreased, it results in high GSNO levels and S-nitrosylated proteins [84]. Changes in the activity of GSNOR determine the whole pool of SNOs and may regulate cell signaling. Moreover, a deficiency in GSNOR is linked with the presence of NSS and tissue damage [85]. This correlates with GAPDH S-nitrosylation that leads to covalent inactivation of the enzyme [86]. This irreversible alteration requires the synthesis of new proteins to reestablish the activity of GAPDH [87].

NO alters histone PTMs, DNA methylation, and miRNA levels [88]. DNA reparation and maintenance of genomic stability depend on the nuclear enzyme PARP-1. In normal conditions, this enzyme participates in DNA base excision repair and in maintaining the genomic stability. However, it can be overactivated by RNS to induce DNA damage. Activation of PARP-1 is mainly triggered by the presence of DNA single-strand breakages, and therefore, it is overactivated when endogenously and exogenously generated ONOO^−^ break the DNA strand [89].

The enzyme also automodifies itself by PARylation. The PARP-1 auto-PARylation represents a major regulatory mechanism [90]. Upon binding to damaged DNA, PARP-1 forms homodimers and catalyzes the cleavage of NAD^+^ into nicotinamide and ADP-ribose to form ADP-ribose polymers and long branches on glutamic acid residues of several target proteins including histones and by PARP-1 automodification. NSS triggers extensive DNA breakage, PARP-1 overactivation, and the consequent depletion of the cellular stores of substrates such as NAD^+^, impairing the Krebs cycle, glycolysis, and mitochondrial electron transport. This results in ATP depletion and the consequent cell dysfunction and death by necrosis [90, 91].

## 5. Oxidative, Reductive, and Nitrosative Damage to Classical Epigenetic Cues

Epigenetic modifications, such as the methylation/demethylation of DNA and histone proteins and histone acetylation/deacetylation can be produced and eliminated by enzymes that consume several metabolites derived from physiological pathways including stress mediators. These metabolites determine the activity of epigenetic enzymes such as methyltransferases, deacetylases and kinases, and histones controlling the chromatin structure that ultimately enhances or reduces gene expression. Therefore, environmental stimuli such as dietary exposure and nutritional status that alter the concentration of metabolites affect epigenetic regulation, including S-adenosylmethionine (SAM), acetyl-CoA, nicotinamide adenine dinucleotide (NAD^+^), flavin adenine dinucleotide (FAD), *α*-ketoglutarate, succinate, fumarate, and ATP [92]. Therefore, the concentration of crucial nutrients, such as glucose, glutamine, and oxygen, spatially and temporally modulates epigenetic modifications to regulate gene expression and the reaction to stressful microenvironments in diseases [93].

Epigenetic changes acquired during life may affect the expression of genes related with the mitochondrial function. Metabolic alterations in mitochondria probably impact on the availability of metabolites for chromatin-modifying enzymes, promoting epigenetic signals that change the chromatin and, therefore, gene transcription. In fact, the availability of some metabolites synthetized in mitochondria, such as ATP, CoA, NADH, and *α*-ketoglutarate, among others, promotes different kinds of epigenetic modifications. Therefore, mitochondrial sensitivity caused by environmental factors and lifestyle changes, like sedentarism, physical activity, overnutrition, and balanced nutrition, may support, or prevent, many of the effects that promote metabolic disorders. Increasing reports show that the damage on mitochondrial DNA plays an important role in disease development [94, 95].

### 5.1. Oxidative Damage to Epigenetic Cues on DNA and Histones

Methylation compacts DNA and inhibits transcription; this process depends on a balance of the activities of methylases and demethylases. There are passive and active DNA demethylation processes. An example of passive demethylation could be a loss of balance between methylation and demethylation. This imbalance could result from a cascade of epigenetic alterations starting with histone modification enzymes and/or altered methyltransferase activity.

ROS regulate local hypermethylation through cytosine methylation and hydroxymethylation [74], which is associated with repression of transcription. The main enzymes involved in DNA methylation are DNA methyltransferases (DNMTs). DNMT1 is recruited by base excision and mismatch repair proteins which recognize 8-oxo-dG in CpG island promoter regions [96, 97]. OS may cause relocalization of DNMTs resulting in hypermethylation of CpG islands and global hypomethylation. In mammalian cells, H_2_O_2_ may activate at least 40 genes [98].

OS also triggers the depletion of *S*-adenosyl methionine (SAM) which is used to transfer a methyl group by DNMTs, acting on cytosine bases in DNA. Another mechanism for DNA hypermethylation by OS is the inhibition of the ten-eleven translocation (TET) methylcytosine dioxygenase enzymes that promote reversal of DNA methylation [99, 100]. Krebs cycle intermediates succinate and fumarate inhibit TET enzymes [101].

In histones, OS does not always influence methylation of lysine marks in the same way. Some lysine residues may be hypermethylated while others may be unaffected or hypomethylated [102]. This may be due to different sensitivities toward oxidation, competitive inhibition, or SAM depletion of the different enzymes or of the microenvironment of the enzyme complex [103]. Hence, specific and local rather than global regulation of histone methyl transferase (HMT) and histone demethylase (HDM) activities might predominate *in vivo*.

In addition to DNA and histone methylation, histone acetylation also produces global changes associated with chromatin relaxation and transcriptional activation. Histone acetylation is controlled by histone acetyltransferases (HATs) and histone deacetylases (HDACs) [104–106]. OS is an important modulator of HDAC function. HDACs are alkylated and inhibited by several reactive aldehydes, which results in changes in gene expression [107, 108]. Furthermore, inhibition of HDACs by OS might confer resistance [109].

Cysteine carbonylation may inactivate a class of HDACs named sirtuins (SIRT) that also have monoribosyltransferase activity; SIRT1 inactivation leads to an increased acetylation and inhibition of the transcription factor forkhead box O-3 (FoxO3) that is a key player in a variety of cellular processes including metabolism, apoptosis, and proliferation [110]. In contrast, activation or overexpression of SIRT1 protects cells from OS during senescence [111, 112]. When SIRT3 is carbonylated, it leads to the upregulation of stress-induced genes [113, 114]. Nuclear SIRT3 is rapidly degraded when cells are exposed to OS.

Other class II HDACs translocate from the nucleus to the cytoplasm in a ROS-dependent manner increasing transcription of myocyte enhancer factor-2- (MEF2-) dependent genes [114, 115]. These transcription factors regulate differentiation and play important roles in stress resistance. Furthermore, ROS can also increase histone deacetylation by directly stimulating HDAC expression despite being potent inhibitors [116, 117] or indirectly enhance HDAC activity [118].

OS also indirectly modifies the global levels of histone phosphorylation. Phosphorylation of histone serine, threonine, and tyrosine residues regulates gene expression, DNA repair, and mitosis [119]. OS results in the formation of DNA double-strand breaks, which lead the phosphorylation of H2AX to trigger DNA repair [120–122]. Oxidation inhibits the histone-targeting protein phosphatases PP1 and PP2A by their catalytic metal ion [123].

OS also induces the formation of methionine sulfoxide from methionine, and this molecule can immediately react with OH^·^ to generate a methyl radical that nonenzymatically and nonspecifically methylates cytosine in the DNA [124]. This phenomenon could produce deleterious effects in the epigenome [125]. Furthermore, it was recently reported that OS affects methionine synthase, an important enzyme in the regeneration of methionine from homocysteine [126].

Although nuclear DNA methylation is well established, mitochondrial DNA methylation is a matter of debate. Even if it had been reported that methylases could not access mitochondria and that mtDNA had no histones, recent evidence has suggested that mtDNA can be epigenetically regulated by methylation [127]. Methylase, 5-methylcytosine (5mC), and 5-hydroxymethylcytosine (5hmC) at CpG dinucleotides have been reported in mitochondria. Recently, a variant of the DNA methylase 1, mitochondrial (mtDNMT1), which uses an upstream alternative translation start site, which might lead to the inclusion of a mitochondrial targeting sequence was described. This mtDNMT1 attaches to the mitochondrial genome in proportion to the density of CpG dinucleotides. Therefore, cytosine methylation in mtDNA may play a role in establishing epigenetic cues [127, 128].

### 5.2. Reductive Stress and Epigenetic Cues

Oxidized GSH inhibits the activity of SAM synthetase and methionine adenosyltransferase 1A (MAT1A). This key enzyme is involved in the synthesis of SAM, which is used by DNMTs and HMTs as a substrate for DNA and histone methylation, respectively [129, 130]. Therefore, it is possible to alter the methylation status of the genome and modify the epigenetic signature in cells by modulating SAM levels. Methylation of DNA and histone constitutes one of the most studied chemical modifications in the epigenetic code, which shapes gene-expression patterns usually, but not always, repressing gene transcription. Interestingly, replenishment of GSH levels recovers the activity of MAT1A [131] thereby contributing to the homeostasis of DNA and histone methylation.

S-Glutathionylation of histone H3 is a PTM in the histone code [132]. In this modification, GSH binds to Cys110 in histone H3 producing changes in the stability of the nucleosomes and altering the chromatin structure by decreasing the proportion of *α*-helices. Interestingly, S-glutathionylation of H3 is increased in proliferating cells but not in quiescent cells, suggesting that GSH modifies the structure of the chromatin during cell proliferation. Furthermore, the ability of GSH to open the chromatin may increase the susceptibility of DNA to the attack of DNA-interacting drugs [133, 134].

GSH may be involved in epigenetic events. There is a GSH-dependent enzymatic mechanism that prevents the production of the methionine sulfoxide induced by OS. The GSH/glutaredoxin system can regenerate the activated form of methionine sulfoxide reductase, the enzyme that converts methionine sulfoxide to methionine [135]. Therefore, GSH/glutaredoxin/MSR prevents the generation of the methyl radical and contributes to the regeneration of methionine, which, in turn, is introduced in the methionine cycle to recover the SAM levels. Glutathionylation also inactivates SIRT1 [136].

H_2_S in plasma decreased with age, and this decrease is associated with a decreased expression of the mRNA of CSE which synthetizes it, while DNMT expressions are increased. In the CSE promoter, transcription was downregulated by enhanced DNA methylation. The expression and activity of DNMT was upregulated by oxidized low-density lipoprotein, and suppression of DNMT reversed the decreases of CSE mRNA [137].

There is also a significant upregulation of CBS which also synthetizes H_2_S. Its mRNA levels are associated with the demethylation of CBS gene in rats injected with *Mycobacterium butyricum*. Promoter DNA hypermethylation is traditionally recognized to repress gene expression. CBS-H_2_S signaling is crucial for inflammatory hyperalgesia and the DNA demethylation of the CBS promoter region.

Furthermore, H_2_S exerts some of its beneficial effects through SIRT1, and treatment with H_2_S donors such as Na_2_S abolishes OS in cardiomyocytes via SIRT upregulation. H_2_S also attenuates inflammation partially by promoting SIRT3 [136].

### 5.3. Nitrosative Stress and Epigenetic Cues

NO synthesis and release are epigenetically controlled, and in turn, NO may act as an epigenetic regulator. However, many of the epigenetic properties of this agent remain unknown [54]. NO inhibits HDAC complexes by enhancing histone acetylation and promotes a chromatin state that supports gene expression. NO might also regulate other targets of redox molecules such as methyltransferases and demethylases [138].

Cell cycle arrest and differentiation are also regulated by epigenetic changes associated with NO. HDACs are intranuclear targets of NO, but, due to the highly diffusible nature of NO, it is possible that many other nuclear factors may be regulated by NO [139].

NO participates in histone PTMs that control histone-modifying enzymes. The capacity of NO to regulate the activities and cellular localizations of these enzymes is due to its capacity to form iron–nitrosyl complexes and S-nitrosothiols that mediate the epigenetic effects of NO [140].

NO diffuses from the cytosol to the nucleus or can be produced directly by the nuclear eNOS. NO may also be generated by ligand-activated receptors and by environmental factors including shear stress. The production of NO leads to the activation of the PI3K/Akt pathway that results in eNOS phosphorylation. Cytosolic NO regulates the translocation and activation of nuclear class II HDACs. It also induces PTMs such as tyrosine nitration and S-nitrosylation of transcription factors. NO may also posttranslationally modify HDAC2 and transcription factors in the nucleus [54, 141].

NSS affects DNA and histone methylation by inhibiting the Jumonji C (JmjC) demethylases. NO inhibits the JmjC domain containing demethylase KDM3A by binding to the catalytic iron [142].

During differentiation, these processes may lead to the repression of stem and nonmesodermal genes and to the activation of vascular genes [141]. NO determines miR-200a, miR-200b, miR-200c, and miR-429 expressions, which induce meso-endoderm and precursor vascular marker expression.

NSS is also a potent modulator of HDAC function. HDAC2 can be nitrosylated having controversial effects [143]. Nitrosylation leads to displacement of HDACs from chromatin to activate gene expression [144]. Moreover, cysteine nitrosylation decreased HDAC2 activity [145]. SIRT1 and SIRT6 can be inactivated through peroxynitrite-mediated nitrosylation, which could be oncogenic [143, 146].

## 6. Induction of Other Possible Epigenetic Modification on Proteins by Oxidative, Reductive, and Nitrosative Stress

### 6.1. Carbonylation and Glycation of Histones Resulting from Oxidative Stress

OS is an epigenetic modulator since ROS induce epigenetic cues such as reactions with reactive aldehydes, glycation of histones, or carbonylation of proteins [74]. Reactive aldehydes that modify histones could constitute epigenetic cues induced by OS since these aldehydes are produced intracellularly in a ROS-dependent way. Highly reactive *α*,*β*-unsaturated aldehydes, such as glyoxal, malondialdehyde, acrolein, 4-hydroxy-2-nonenal (4-HNE), or 4-oxo-2-nonenal (4-ONE), are enzymatically or non-enzymatically produced by lipid peroxidation [147]. Aldehydes readily react with proteins to form carbonyl adducts.

ROS also promote the production of the glucose metabolites, methylglyoxal and 3-deoxyglucosone [48, 148]. These carbonyl species interact with cysteines, arginines, lysines, or histidines in histones resulting in AGEs or advanced lipoxidation end products thus elevating protein cross-linking and may constitute epigenetic cues [149]. Histones are the common targets of AGEs and lipoxidation end products [150]. When histones H1, H2A, and H3 are altered with 3-deoxyglucosone, they turn less thermostable resulting in partial unfolding which may cause alterations in chromatin structure and gene expression [151–153].

Another PTM resulting from OS is carbonylation. When histones are carbonylated, they may disappear from the chromatin, diminishing nucleosome content since irreversibly damaged histones are removed by the nuclear proteasome [154, 155]. A global reduction of chromatin-associated H3 correlates with increased transcription at histone-depleted loci [156]. Stabilization of histones has also been reported in *in vitro* studies.

### 6.2. Glutathionylation of Histones Resulting from Reductive Stress

There is an association between GSH metabolism and the control of epigenetic mechanisms at different levels such as substrate availability, enzymatic activity for DNA methylation, and changes in the expression of microRNAs and histones. The molecular pathways by which GSH can control epigenetic events remain unknown; however, the role of GSH in the epigenetic mechanisms is through structural alterations and probably through other pathways. Mutations in enzymes involved in GSH metabolism and the alterations of the levels of cofactors affecting epigenetic mechanisms may explain the link between GSH and the establishment of epigenetic cues [157].

Reductive stress causes S-glutathionylation of proteins, and this reaction may constitute an epigenetic cue. Glutathione-S-transferases (GSTs) can glutathionylate cysteines that have been oxidized to sulfenic acid using GSH. This reaction protects residues from further, irreversible oxidation to sulfonic acid [158, 159]. However, OS also increases the oxidation of GSH to GSSG, decreasing the GSH/GSSG ratio. Histone H3, which is one of the basic proteins in the nucleosomes, is S-glutathionylated giving rise to gamma-L-glutamyl-L-cysteinylglycine [157]. Increased glutathionylation also correlates with higher GSH levels and drug resistance [134].

Histone H3 is glutathionylated in proliferating cells. Glutathionylation of H3 decreases nucleosome stability and facilitates gene expression and DNA replication [79]. It is conceivable that an OS-induced reduction in glutathionylation may protect cells from OS by leading to chromatin compaction and inhibition of replication of potentially damaged DNA. Furthermore, reduced histone glutathionylation might contribute to global gene regulation.

### 6.3. Epigenetic-Like Nitration of Histones Resulting from Nitrosative Stress

*In vitro* exposure of recombinant histones H1 and H3 to peroxynitrite leads to tyrosine nitration. Nitrated histones show an increase in structured domains, specifically *β*-sheet structures, and increased thermostability. This nitration might contribute to protection of DNA from oxidative damage during OS [160, 161].

ONOO^−^ can cause nitration of tyrosine residues in intracellular proteins. Nitration of tyrosine involves incorporation of the ONOO^−^ and ^·^NO_2_ production by heme proteins [162–164]. 3-Nitrotyrosine may damage proteins or render them less active. Nitrotyrosine has been considered an index of RNS formation [165, 166].

## 7. Oxidative, Reductive, and Nitrosative Stress Effects on Epigenetic Cues Participating in Cardiometabolic Diseases

The three types of stress have been implicated in the pathophysiology of many disorders including metabolic and cardiovascular diseases. In the next sections, we will describe their participation in obesity, metabolic syndrome, and diabetes; in atherosclerosis and cardiomyopathy; and in endothelial dysfunction and hypertension. We discuss them in separate paragraphs even if many of the mechanisms are common to some of them.

### 7.1. Obesity, Metabolic Syndrome, and Diabetes

Obesity is an important risk factor for cardiovascular diseases and has been associated with inflammatory conditions. White adipose tissue is recognized as an essential immunoendocrine organ that controls energy balance and metabolism. Adipocytes play pivotal roles through the secretion of a variety of adipokines that are implicated in metabolic disorders.

Leptin is a pleiotropic adipokine whose plasma concentration is generally proportional to adipose mass and is involved in the regulation of food intake, in immune and inflammatory responses, and in cell proliferation, among other functions [167]. However, leptin may also have adverse effects such as the induction of OS by activating NADPH oxidase and the activation of iNOS that leads to NSS through production of peroxynitrite resulting in protein nitration. These reactions contribute to the activation of inflammatory pathways [168]. These mechanisms have been identified in steatohepatitis, an obesity-associated pathology [169].

Leptin also decreases glyceroneogenesis and fatty acid reesterification, therefore resulting in fatty acid release by white adipose tissue. This effect is mediated by the nitration of the cytosolic isoform of phosphoenolpyruvate carboxykinase (PEPCK-C), the key enzyme of glyceroneogenesis and in hepatic gluconeogenesis [170].

Regarding epigenetic control in obesity, SIRT2 is the most abundant sirtuin in adipocytes from white and brown adipose tissues [171]. SIRT2 expression was decreased in white adipose tissue from rats with metabolic syndrome and might promote fat accumulation [172]. SIRT3 is the major mitochondrial deacetylase regulating mitochondrial metabolism, adaptive thermogenesis, energy homeostasis, and apoptosis and is decreased in obese mice [173]. Moreover, SIRT3 plays an important role in adaptive thermogenesis of brown adipose tissue regulating uncoupling protein one (UCP-1), peroxisome proliferator-activated receptor gamma coactivator 1-alpha (PGC-1*α*), cytochrome c oxidase, and ATP synthase expression. White adipose tissue from control and metabolic syndrome rats expressed SIRT3 in the same proportion [172].

Epigenetic cues importantly participate in the development of metabolic syndrome. SIRT1, which is an important regulator of hepatic glucose metabolism, is underexpressed in rats having metabolic syndrome. This sirtuin improves insulin signaling and promotes fatty acid metabolism [174, 175]. However, SIRT1 overexpression does not have a significant effect on adipokine secretion in a metabolic syndrome rat model [172]. SIRT1 is also increased in the aortas from metabolic syndrome rats and may be responsible for hypertension related to this metabolic disorder [176].

In diabetes, where hyperglycemia is present, high glucose levels induce an excessive O_2_^−^ production which may constitute a unifying link for the development and progression of diabetes together with its micro- and macrovascular complications. OS and the activation of the antioxidant defense systems precede and constitute a consequence of the development of the main diabetic complications including diabetic coronary atherosclerosis. In diabetes and coronary atherosclerosis, there are epigenetic changes such as DNA methylation and histone PTMs that modify the chromatin accessibility to transcriptional regulatory proteins. These epigenetic changes alter transcriptional programs to initiate atherogenic and inflammatory phenotypes [177].

### 7.2. Atherosclerosis and Cardiomyopathy

Atherogenesis is accelerated when there is an imbalance between the antioxidant capability activity and ROS and cells may be injured due to oxidation of DNA and cellular proteins including histones and reparation proteins and of lipids. It also activates cell death signaling pathways [178].

Epigenetic modulators are importantly involved in the control of vascular, immune, and tissue-specific gene expression in the atherosclerotic lesion. Human atherosclerotic lesions display hypomethylation of genomic DNA. These epigenetic mechanisms change the accessibility of chromatin by DNA methylation and histone modifications. There are also changes in methylation in promoter regions of several genes that participate in the pathogenesis of these disorders including the gene of extracellular superoxide dismutase, the estrogen receptor-*α*, the eNOS, and 15-lipoxygenase [179]. There is also an important association between inflammation and reprogramming of the epigenome [178].

Epigenetic changes may also be related to the pathogenetic features of diseases, such as smooth muscle cell hyperproliferation, accumulation of lipids, and modulation of immune responses [179].

In monocyte-/macrophage-mediated inflammation and atherosclerosis, there is deregulation of the CSE-H_2_S pathway through the epigenetic alterations on DNA methylation that leads to an inflammatory disorder [137].

H_2_S is involved in inflammation where it alters the expression and activity of DNMTs [180]. In some tissues, the exposure to H_2_S results in apoptosis [181]. Moreover, H_2_S may react with the reactive oxygen/nitrogen species produced under inflammatory conditions [182]. It has a protective role against cellular damage by inflammation, and it also participates in angiogenesis, cytoprotection, nociception, stimulation of ATP-sensitive potassium ion channels, myocardial contractility, vascular tone, blood pressure, and ischemia-reperfusion [183].

The epigenetic alterations found in atherosclerosis explain, in part, the dietary effects on this disease. Since epigenetic processes are reversible, they may provide an excellent therapeutic target for therapies directed toward modification of the epigenetic status of vascular cells which might constitute new tools to control atherosclerosis-related cardiovascular diseases.

Hypertrophic cardiomyopathy (HC) is characterized by RS, protein aggregation, and heart failure in transgenic mice. HC is characterized by excessive ubiquitination by activation of Nrf2. Pathological hypertrophy and remodeling are induced by Nrf2 in the heart. Nrf2 deficiency is linked to GSH depletion *in vivo* and *in vitro* which in turn prevents RS in the myocardium [184].

Development of cardiac hypertrophy and heart failure causes deregulation of GSH homeostasis that leads to RS and Nrf2 activation in heart failure. Mutant protein aggregation in cardiomyopathy is initially due to ROS generation and then maintained by keap1 dysfunction through its sequestration into the protein aggregates. Thus, activation by nuclear translocation of Nrf2 is sustained leading to continuous upregulation of the transcription of antioxidant enzymes contributing to RS [185]. Increased and sustained activation of Nrf2 leads to RS where the reductive capacity of the cell and/or the concentration of reducing equivalents increase the levels of GSH and NADPH, exceeding ROS production [186]. Excess activation of Nrf2 is also associated with a variety of other cardiac pathologies [187].

In addition, ONOO^−^ inhibits the mitochondrial respiratory chain and triggers apoptosis at the subcellular level in cardiomyocytes [188].

In cardiomyopathy, there is a decrease in proper protein ubiquitination and degradation due to RS which causes intracellular oxidative modifications [189].

### 7.3. Endothelial Dysfunction and Hypertension

Vascular tissues express abundant enzymes producing H_2_S such as CSE, CBS, and 3MST and therefore produce a large amount of H_2_S that participates in vascular modulation [190]. Deficiency of CSE reduces H_2_S production in vascular tissues leading to endothelial dysfunction and high blood pressure in an age-dependent manner [191].

The reparation of DNA damage is fundamental to normal cell development and replication, and H_2_S attenuates DNA damage in human endothelial cells and fibroblasts by S-sulfhydration of MEK1. H_2_S also protects vessels from cellular aging. When it reaches the nucleus, it may inhibit the proliferation of vascular smooth muscle cells by epigenetic mechanisms involving inhibition of the transcription and expression several transcription factors [191].

ONOO^−^ increase is associated with a reduced PARP-1 pathway which contributes to the endothelial dysfunction [192]. *In vitro*, DNA damage and PARP-1 activation occur in endothelial cells exposed to various ROS and the ONOO^−^ infusion in isolated perfused hearts resulting in severe impairment of the endothelial-dependent relaxation [193, 194].

Hypertension is associated with other pathologies such as diabetes, metabolic syndrome, and obesity, and the population may be predisposed to develop it by the genetic background and unhealthy lifestyles. An elevated sucrose ingestion which leads to OS has been associated to the development of hypertension. We have previously published that the administration of sucrose during a short period near weaning in rats (postnatal days 12 to 28) increases the risk of developing hypertension when the organisms reach adulthood and that this elevation in blood pressure is accompanied by OS [195, 196].

Regarding arterial essential systemic hypertension, the epigenetic cues that link it with OS at the vascular level have also been described [6, 197]. Sirtuins may play an important role as epigenetic cues for the development of hypertension [198]. SIRT1 increases and promotes the activity of coupled eNOS, and SIRT3 activates SOD, which has an antioxidant capacity [200]. SOD reduces the ROS that uncouple eNOS and increase NO levels [199]. SIRT3 was found to be decreased at the end of a short period near weaning when rats received sucrose [195, 196].

In hypertension, H_2_S-producing enzymes CBS, CSE, and 3MST are diminished. In the kidney vasculature, CBS, CSE, and 3MST enzymes constitute prime targets of OS and NSS, leading to a decrease in H_2_S concentration [192].

Angiotensin II (Ang II) can induce through PARP-1 activation the formation of protein 3-nitrotyrosine, eNOS uncoupling, tetrahydrobiopterin (BH_4)_ reduction, and DNA breakage [200]. The Ang II-PARP-1 pathway is present in endothelial dysfunction, in human diabetes, and in a rat model with essential hypertension [201]. There is also evidence that Ang II may induce NSS in peripheral organs and blood vessels [202]. In addition, Ang II can directly activate NFĸB and/or indirectly through induction of O_2_^−^ production, which leads to iNOS induction, resulting in NO and ONOO^−^ overproduction. ONOO^−^ overproduction results in BH_4_ oxidation and leads to uncoupling of eNOS [203]. However, BH_4_ supplementations might reduce the vascular damage by Ang II thereby preventing uncoupling of eNOS and decreasing NSS [204].

## 8. Conclusions

OS, RS, and NSS are interrelated since RS results from an overactivation of antioxidant systems and NSS is the result of the overactivation of the oxidation of NO. OS, RS, and NSS, acting alone or through their interaction, result in damage to the DNA structure, causing strand breaks and the formation of 8-oxo-dG. The three types of stress modify histones and enzymes that determine epigenetic cues (DNA methyl transferases, histone acetyltransferases, and deacetylases) by posttranslational mechanisms. Some of these changes are shown in Table 1.

The three types of stress also alter intracellular signaling pathways and regulate the activity of DNA reparation enzymes. The posttranslational alterations they produce include reactions such as carbonylation, glycation, glutathionylation, sulfhydration, nitration, and nitrosylation. Furthermore, the three types of stress may induce the establishment of new epigenetic marks and may impair the reparation mechanisms of DNA damage. These changes are summarized in Table 2.

The changes induced by OS, RS, and NSS on epigenetics could underlie cardiometabolic diseases including obesity, metabolic syndrome, diabetes, endothelial dysfunction, hypertension, atherosclerosis, and hypertrophic cardiomyopathy as discussed in this review. The development of cardiometabolic disorders in adult life may be programed since early stages of development by epigenetic cues which may be established or modified by OS, RS, and NSS.

Therefore, OS, RS, and NSS importantly participate in the mediation of the impact of the early life environment on later health heritability. These modifications depend on the union and release of chemical residues on a DNA sequence and/or on amino acid residues in histones, and therefore, they are reversible and potentially treatable. Epigenetic cues importantly participate in the development of cardiometabolic diseases.

## Figures and Tables

**Table 1:** Changes induced by OS, RS, and NSS on DNA and associated proteins and on proteins that establish classical epigenetic cues.

Effects on DNA and associated proteins	OS	-Activation of DNMTs [74, 98]
		-Depletion of SAM [99–101]
		-Inhibition of TET enzymes [99–101]
		-Methylation through formation of methionine sulfoxide [124–126]
	RS	- Activation of DNMT by H_2_S [137]
		- Inhibition of SAM by GSH [129, 130]
		-Decreased production of methionine sulfoxide [135]
		-Damage to DNA repair mechanisms [23, 50]
	NSS	-Inhibition of HDAC by NO [138]
		-Inhibition of JmjC demethylases [142]
		-Activation PARP-1 [23, 50]
Effects on proteins that establish epigenetic cues	OS	-Controversial effects on methylation of lysines by HMT [102]
		-Increased acetylation through inhibition of HDAC [107–109]
		-Increased acetylation through inactivation of SIRT1 [111, 112]
		-Degradation of SIRT3 [113, 114]
		-Increased phosphorylation [119]
	RS	-Glutathionylation of H3 causing instability of the nucleosome [133, 134]
		-Inactivation of SIRT1 by GSH [136]
		-Upregulation of SIRT3 by H_2_S [136]
	NSS	-Increased histone acetylation by NO [138]
		-Controversial effects on HDAC [143]
		-Inactivation of SIRT1 and 6 [143, 146]

OS: oxidative stress; RS: reductive stress; NSS: nitrosative stress; DNMT: DNA methyltransferase; SAM: S-adenosyl methionine; TET enzymes: ten-eleven translocation (TET) methylcytosine dioxygenases; GSH: glutathione; H_2_S: sulfhydric acid; HDAC: histone deacetylase; NO: nitric oxide; JmjC: Jumonji C; PARP1: poly [ADP-ribose] polymerase 1; HMT: histone methyl transferase; SIRT: sirtuin deacetylase.

**Table 2:** Establishment of new nonclassical epigenetic cues by OS, RS, and NSS.

Type of stress	Mechanism	Effect
OS	Formation of AGEs and advanced lipoxidation end products	Elevated cross-linking which could act as an epigenetic cue [149–153]
	Carbonylation	Loss of histones that leads to increased transcription [154–156]
RS	Alterations in GSH metabolism	Decreased nucleosome stability that facilitates gene expression and DNA replication [157–159]
NSS	Nitration of tyrosine residues	Protection of DNA against oxidative damage [160, 161]

OS: oxidative stress; RS: reductive stress; NSS: nitrosative stress; AGEs: advanced glycation end products; GSH: glutathione.
